# SNP-based analysis of genetic diversity in anther-derived rice by whole genome sequencing

**DOI:** 10.1186/1939-8433-6-6

**Published:** 2013-03-14

**Authors:** In-Seon Jeong, Ung-Han Yoon, Gang-Seob Lee, Hyeon-So Ji, Hyun-Ju Lee, Chang-Deok Han, Jang-Ho Hahn, Gynheung An, Tae-Ho Kim

**Affiliations:** Rural Development Administration, Genomics Division, National Academy of Agricultural Science, Suwon, 441-707 Republic of Korea; Department of Biochemistry, Gyeongsang National University, Jinju, 660-701 Republic of Korea; Department of plant molecular systems biotechnology and Crop biotech institute, Kyung Hee university, Yongin, 446-701 Republic of Korea

**Keywords:** Korean rice, Anther culture, Re-sequencing, Genetic diversity

## Abstract

**Background:**

Anther culture has advantage to obtain a homozygous progeny by induced doubling of haploid chromosomes and to improve selection efficiency for invaluable agronomical traits. Therefore, anther culturing is widely utilized to breed new varieties and to induce genetic variations in several crops including rice. Genome sequencing technologies allow the detection of a massive number of DNA polymorphism such as SNPs and Indels between closely related cultivars. These DNA polymorphisms permit the rapid identification of genetic diversity among cultivars and genomic locations of heritable traits. To estimate sequence diversity derived from anther culturing, we performed whole-genome resequencing of five Korean rice accessions, including three anther culture lines (BLB, HY-04 and HY-08), their progenitor cultivar (Hwayeong), and an additional japonica cultivar (Dongjin).

**Results:**

A total of 1,165 × 10^6^ raw reads were generated with over 58× coverage that detected 1,154,063 DNA polymorphisms between the Korean rice accessions and *Nipponbare*. We observed that in Hwayeong and its progenies, 0.64 SNP was found per one kb of *Nipponbare* genome, while Dongjin, bred by a conventional breeding method, had a lower number of SNPs (0.45 SNP/kb). Among 1,154,063 DNA polymorphisms, 29,269 non-synonymous SNPs located on 30,013 genes and these genes were functionally classified based on gene ontology (GO). We also analyzed line-specific SNPs which were estimated 1 ~ 3% of the total SNPs. The frequency of non-synonymous SNPs in each accession ranged from 26 SNPs in Hwayeong to 214 SNPs in HY-04.

**Conclusions:**

The genetic difference we detected between the progenies derived from anther culture and their mother cultivar is due to somaclonal variation during tissue culture process, such as karyotype change, chromosome rearrangement, gene amplification and deletion, transposable element, and DNA methylation. Detection of genome-wide DNA polymorphisms by high-throughput sequencer enabled to identify sequence diversity derived from anther culturing and genomic locations of heritable traits. Furthermore, it will provide an invaluable resource to identify molecular markers and genes associated with diverse traits of agronomical importance.

**Electronic supplementary material:**

The online version of this article (doi:10.1186/1939-8433-6-6) contains supplementary material, which is available to authorized users.

## Background

Advances in genome sequencing technologies have aided in the discovery of millions of genome-wide DNA polymorphisms, single nucleotide polymorphisms (SNPs) and insertion-deletions (InDels). These are invaluable resources in analyzing genetic diversity in a population and in establishing the linkage relationship between genomes and heritable traits (Chen et al. 2011; Osman et al. 2003). Reference genome sequences for several crop species are now available, which permits both rapid identification of candidate genes through bioinformatic analysis and SNP discovery through comparison of the reference sequence with ones of various cultivars (Edwards and Batley 2010; Kim et al. 2010).

SNPs are the most common polymorphisms in the genomes of most organisms and are important molecular markers in genetic research for marker-assisted breeding (Ganal et al. 2009; Jena and Mackill 2008; McCouch et al. 2010; Silva et al. 2012. Since the rice genome was recently sequenced with high accuracy using a japonica rice cultivar, *Nipponbare* (IRGSP 2005), discovering massive numbers of SNPs by comparison with the *Nipponbare* reference sequence has become an effective tool. Recently, whole genome resequencing of rice cultivars using *Nipponbare*, as a reference have been performed using high-throughput sequencers. The whole genome resequencing of the japonica rice cultivar Koshihikari, which is closely related to *Nipponbare* has been completed (Yamamoto et al. 2010). In total 67,051 SNPs have been identified by a comparison between these two genomes. Historical representative rice cultivars were also analyzed to understand the dynamics of genome compositions using typing arrays based on SNPs. In a landrace cultivar of japonica rice 168,228 DNA polymorphisms were discovered by whole genome resequencing, and InDels were also validated by actual use as DNA markers (Arai-Kichise et al. 2011). For identifying agronomically importance genes, the resequencing 50 accessions of cultivated and wild rice revealed 6.5 million high-quality SNPs and identified thousands of genes with significantly lower diversity based on obtained SNPs. These candidate genes were considered to be selected during domestication (Xu et al. 2011).

Anther culturing has the advantages of producing homozygous progeny by induced doubling of haploid chromosomes and the improved selection efficiency for important agronomical plant traits (Janhe et al. 1991). Anther culturing, therefore, has been used as an efficient method to improve agronomically important crops such as rice and barely by producing useful cultivars (Barchi et al. 2010; Kasha and Kao 1970; Kozik et al. 2002; Zagorska et al. 2004). It has been reported that a number of variants have been detected in anther culture lines in several crops including rice (Bairu et al. 2011; Doğramaci-Altuntepe et al. 2001; Evans 1989; Reed and Wernsman 1988; Roy and Mandal 2005; Yan et al. 1996). However, the origins and extents of mutations are not well understood.

In this study, we performed whole genome sequencing to understand the extent of the sequence variation between an anther culture progenitor, Hwayeong, and its progeny lines (BLB, HY-04, and HY-08), which exhibited new agronomically important traits. Also, Dongjin, which is an elite cultivar in Korea, was resequenced to estimate the difference in genomic sequences between a cultivar developed from anther culturing and a cultivar developed by a conventional breeding method. Further genetic research will link sequence diversity with genic factors involved in anther culturing techniques. Also, this study confirms the idea that anther cultures provide valuable resources for developing genetic diversity and for breeding in rice.

## Results

### Sequencing and mapping of the reads to the *Nipponbare* genome

We performed whole genome resequencing of five Korean rice accessions including three anther culture lines (BLB, HY-04 and HY-08), their progenitor cultivar (Hwayeong), and an additional Korean japonica rice cultivar (Dongjin). The sequencing results yielded 118,243 × 10^6^ bps (corresponding to 1,165 × 10^6^ reads) and, on average, 61× coverage of the *Nipponbare* reference genome. The raw reads, which were high quality with Phred Quality Value +33 (> Q20), were used to analyze genetic variations in these five accessions (average 89.9% of total reads).

We mapped a large number of short reads from each of the five Korean rice accessions on to genomic sequences of japonica rice cultivar, *Nipponbare*. The mapping ratio which is a portion of reads that uniquely mapped onto *Nipponbare* genome in different accessions varied from 87% (207 × 10^6^ out of 237 × 10^6^ reads) in HY-04 to 89% (197 × 10^6^ out of 220 × 10^6^ reads) in Dongjin (Table 1). The final effective mapping depth averaged > 54× across the whole genome, with a sequencing depth ranging from 53× in Dongjin to 55× in HY-08. The uniquely mapped reads covered approximately 94% of the *Nipponbare* genome in all five accessions (Table 1). Among chromosomes, chromosome 11 had the lowest ratios, > 12% and > 10% lower, respectively, than the average ratio, in both the genome coverage and mapping depth. All three lines (HY-04, HY-08 and BLB) that were regenerated from anther cultures had the highest ratios of coverage, > 99%, on chromosome 5 and depths from 62× to 68×, which was approximately 10% higher than average, on chromosome 10. In Dongjin and Hwayeong, the highest ratio of coverage was similar to the three anther culture lines on chromosome 5, but chromosome 9 had the highest ratio of depth. However, there was little difference among the five accessions.

**Table 1:** **Reference assembly of each accession onto** ***Nipponbare*** **genome**

	# of reads^a^	Mapped reads		Depth	All mapped nucleotide (bp)	Coverage (%)	3+ mapped nucleotide^b^(bp)	Coverage (%)
		(#)	(%)					
Dongjin	220,275,464	197,217,131	89.53	53.19	365,559,219	95.5	363,351,121	94.92
Sub total	220,275,464	197,217,131	89.53	53.19	365,559,219	95.5	363,351,121	94.92
Hwayeong	233,203,421	206,036,766	88.35	54.36	364,116,034	95.12	361,752,130	94.5
BLB	236,387,205	208,936,303	88.39	55.13	364,684,871	95.27	362,481,239	94.7
HY-04	237,106,362	207,858,683	87.66	54.85	363,086,064	94.85	360,729,934	94.24
HY-08	238,445,614	210,468,066	88.27	55.53	363,324,672	94.92	360,872,314	94.27
Sub total	945,142,602	833,299,818	352.67	219.87	1,455,211,641	380.16	1,445,835,617	377.71
Sub average	236,285,651	208,324,955	88.17	54.97	363,802,910	95.04	361,458,904	94.43
Total	1,165,418,066	1,030,516,949	442.20	273.06	1,820,770,860	475.66	1,809,186,738	472.63
Average	233,083,613	206,103,390	88.44	54.61	364,154,172	95.13	361,837,348	94.53

a: the number of reads which were generated by high-throughput sequencer.

b: base pair of nucleotide which mapped over 3 reads on one site.

### Detection of DNA polymorphisms

**Figure 1:**
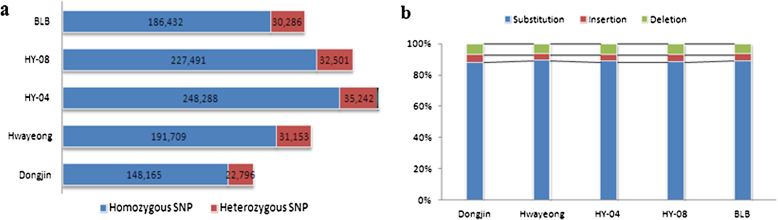
**Distribution of SNP types.** (**a**) Homozygous and heterozygous SNPs from each accession. Homozygous SNPs accounted for approximately 87% of the total potential SNPs. (**b**) The ratio of SNP types. Substitutions, insertions and deletions were 88.7%, 4.5%, and 6.6%, respectively, among DNA polymorphisms.

The total number of DNA polymorphisms was 1,154,063 including 1,024,202 SNPs, 53,180 insertions and 76,681 deletions between the five accessions and the Nipponbare genome (Figure 1b). On average, 230,813 SNPs per accession were detected, which means that 0.6 SNP was found per one kb of Nipponbare genome (382 Mb). We observed that all accessions had similar results among the DNA polymorphisms with 88.7% being substitutions, 4.5% being insertions, and 6.6% being deletions (Figure 1b).

Averages of 245,776 DNA polymorphisms were detected within Hwayeong, BLB, HY-04 and HY-08. All these lines, including Hwayeong, were developed via anther cultures. There were larger DNA polymorphisms in Dongjin, which was bred by a conventional breeding method. HY-04 and HY-08, which have a high yielding ability trait, had slightly higher ratios (> 1.5%) of SNPs than BLB. They showed higher frequencies of substitutions but lower frequencies of InDels than Hwayeong and BLB. The total number of SNPs varied across on each chromosome. Over 50% of Dongjin’s SNPs were located on chromosome 11 and 12 while over 50% of Hwayeong and its anther culture derived lines SNPS were located on chromosome 8 and 11 (Table 2). There were indications of a sequence difference between Hwayeong and its anther culture derived progenies. Hwayeong had its lowest ratio of SNPs (2% of the total) on chromosome 6, but for the three progeny lines the lowest ratio of SNPs was on chromosome 5 (1 to 2%). Potential SNPs were classified into two types, homozygous and heterozygous SNPs, based on the mismatch frequency with *Nipponbare* when there were more than two bases in the identity position. Approximately 87% of the SNPs from all five of the accessions were homozygous and 13% were heterozygous (Figure 1a).

**Table 2:** **The number of SNPs on individual chromosomes detected between each accession and** ***Nipponbare***

	Dongjin		Hwayeong		HY-04		HY-08		BLB	
	# of SNPs	SNP/100 kb	# of SNPs	SNP/100 kb	# of SNPs	SNP/100 kb	# of SNPs	SNP/100 kb	# of SNPs	SNP/100 kb
Chromosome 1	4,126	(9.16)	5,318	(11.81)	30,700	(68.16)	23,902	(53.07)	10,242	(22.74)
Chromosome 2	4,805	(13.06)	7,143	(19.41)	9,399	(25.55)	7,875	(21.40)	9,369	(25.46)
Chromosome 3	1,990	(5.33)	4,318	(11.57)	12,532	(33.59)	12,688	(34.00)	8,152	(21.85)
Chromosome 4	14,197	(39.37)	14,637	(40.59)	14,110	(39.13)	14,505	(40.22)	11,252	(31.20)
Chromosome 5	9,615	(31.97)	4,943	(16.44)	3,782	(12.58)	3,782	(12.58)	5,041	(16.76)
Chromosome 6	7,441	(23.16)	4,263	(13.27)	7,881	(24.53)	3,880	(12.08)	7,158	(22.28)
Chromosome 7	18,667	(61.49)	18,025	(59.38)	20,982	(69.12)	18,548	(61.10)	12,143	(40.00)
Chromosome 8	9,663	(33.87)	70,295	(246.39)	69,329	(243.00)	71,301	(249.92)	66,592	(233.41)
Chromosome 9	3,093	(12.94)	5,864	(24.54)	14,166	(59.28)	14,583	(61.03)	5,743	(24.03)
Chromosome 10	9,799	(41.34)	8,752	(36.92)	22,221	(93.75)	8,704	(36.72)	12,149	(51.25)
Chromosome 11	39,285	(125.83)	63,387	(203.04)	62,336	(199.67)	63,652	(203.88)	59,631	(191.00)
Chromosome 12	48,280	(174.43)	15,917	(57.51)	16,092	(58.14)	16,572	(59.87)	9,246	(33.40)
total	170,961	(47.66)^a^	222,862	(61.74)	283,530	(77.21)	259,992	(70.49)	216,718	(59.45)

a: average DNA polymorphism per 100 kb.

### Annotation of SNPs and InDels

**Figure 2:**
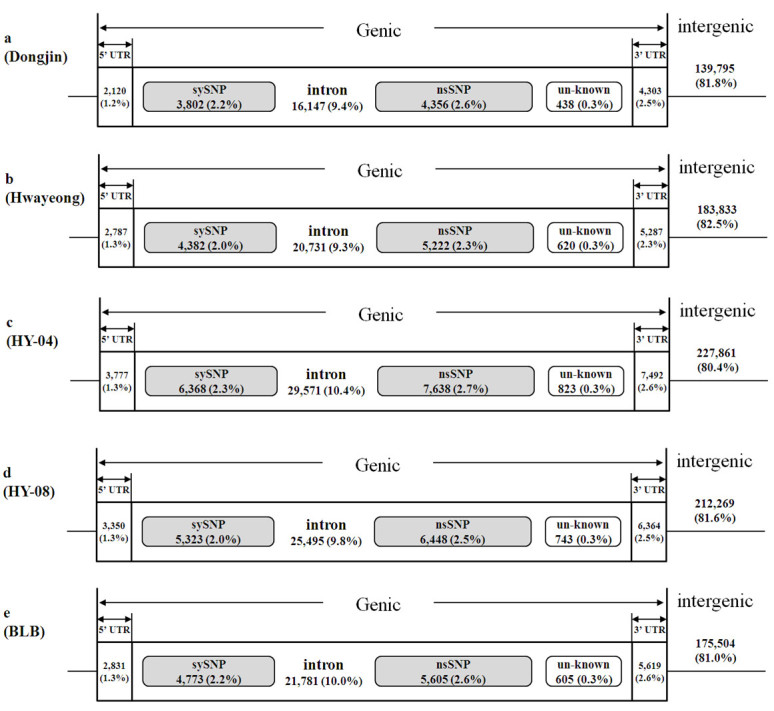
**Classification of SNPs based on locations.** Based on the annotation of IRGSP, SNPs and InDels, SNPs were classified as genic or intergenic. Depending on intra-genic locations, ‘genic’ was further separated into CDS, intron, and UTRs. The number and ratio of SNPs in each class is shown. Unknown SNPs were located in the coding region but were not annotated in IRGSP. (**a**) Dongjin is an elite cultivar in Korea. (**b**) Hwayeong is a mother cultivar of HY-04, HY-08, and BLB. (**c**), (**d**), and (**e**) were derived from Hwayeong via anther culturing.

The Rice Annotation Project Database (RAP-DB) was used to locate the 1,154,063 DNA polymorphisms detected between all five accessions and the *Nipponbare* genome. Accordingly, the total 214,799 SNPs (including InDels, 18.6% of the total) out of 1,154,063 SNPs were found in a gene region, but only 57,146 SNPs (4.95% of the total) occurred in a coding region (Figure 2). Altogether, 29,269 non-synonymous SNPs (2.54% of the total) detected in all five accessions were located in 30,013 genes (Table 3). Among the 42,088 genes annotated with RAP-DB, HY-04 contained the highest number of SNP containing genes. HY-04 carried SNPs in 7,507 genes (17.8% of the total genes) and HY-08 had SNPs in 6,558 genes (15.6% of the total genes) (Table 3). The annotation of SNPs in each of the five accessions revealed that the number of SNPs per gene ranged from 6.61 in Dongjin to 7.42 in HY-04, with a mean of 7.16. Similarly, the number of non-synonymous SNPs per gene ranged from 0.92 in Dongjin to 1.02 in HY-04 (Table 3). On average, the ratio of non-synonymous to synonymous SNPs was 1.16 in the five accessions (Table 3), which is similar to that found in a previous study (McNally et al. 2009). The ratio is higher than that of Arabidopsis (0.83) (Clark et al. 2007) but lower than that of soybean (1.61) (Lam et al. 2010).

**Table 3:** **Distribution of SNPs within genic regions**

Cultivar	Gene		Total SNP in gene			Non-synonymous		Synonymous		Non-synonymous SNP/synonymous SNP
	(/Total gene #)		Count (/total SNP)		SNP/gene	Count	NS/gene	Count	SY/gene	
Dongjin	4,713	11.2%	31,166	18.2%	6.61^a^	4,356	0.92^b^	3,802	0.81^c^	1.14
Sub total	4,713	11.2%	31,166	18.2%	6.61	4,356	0.92	3,802	0.81	1.14
Sub average	4,713	11.2%	31,166	18.2%	6.61	4,356	0.92	3,802	0.81	1.14
Hwayeong	5,471	13.0%	39,029	17.5%	7.13	5,222	0.95	4,382	0.80	1.19
HY-04	7,507	17.8%	55,668	19.6%	7.42	7,638	1.02	6,368	0.85	1.19
HY-08	6,558	15.6%	47,722	18.4%	7.28	6,448	0.98	5,323	0.81	1.12
BLB	5,764	13.7%	41,214	19.0%	7.15	5,605	0.97	4,773	0.83	1.17
Sub total	25,300	60.1%	183,633	18.7%	7.26	24,913	0.98	20,846	0.82	4.67
Sub average	6,325	15.0%	45,908	18.7%	7.26	6,228	0.98	5,212	0.82	1.16
Total	30,013	71.3%	214,799	18.6%	7.16	29,269	0.98	24,648	0.82	5.81
Average	6,003	14.3%	42,960	18.6%	7.16	5,854	0.98	4,930	0.82	1.16

Total gene count (build 5): 42,088.

a: SNP/Gene : Total SNP frequency in each gene (including CDS, intron, UTR region), 31,166/4,713=6.61.

b: NS/Gene : non-synonymous SNP frequency in each gene, 4,356/4,713=0.92.

c: SY/Gene : Synonymous SNP frequency in each gene, 3,802/4,713=0.81.

### Comparison analysis between detected SNPs and dbSNP

**Figure 3:**
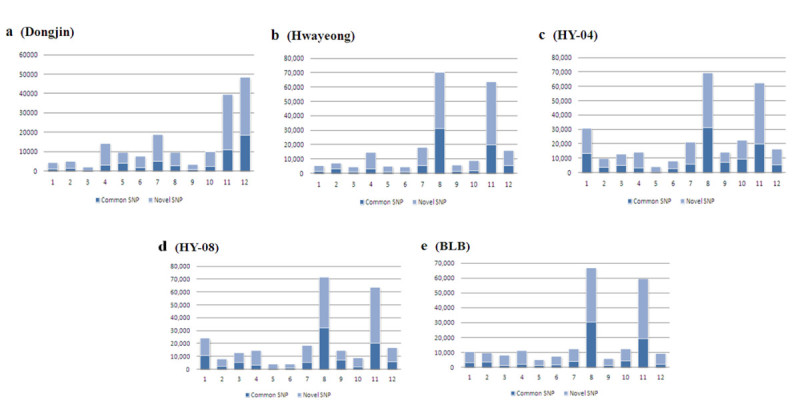
**Comparisons between novel SNPs and dbSNP.** By comparing with NCBI’s dbSNP, SNPs were classified into novel and common. Novel SNPs were more abundant than common ones. In the graph, the x-coordinate and y-coordinate represent each chromosome and the number of SNPs, respectively. (**a**) – (**e**) are the same as described in Figure 2.

We also analyzed whether the detected SNPs were novel SNPs or SNPs reported on the NCBI’s dbSNP. The highest percentage of novel SNPs was shown in Dongjin with only 29.48% common SNPs and 70.52% novel SNPs. The ratio of novel SNPs in HY-04 and HY-08 were nearly 4% lower than Hwayeong and BLB. In HY-04 and HY-08, chromosome 9 had the least difference between common SNPs and novel SNPs at 3.83% and 2.72%, respectively. In Hwayeong and BLB, chromosome 8 showed the least difference between common SNPs and novel SNPs (Figure 3). In contrast, the largest differences between the two SNP types were found on chromosome 5 of HY-04 and HY-08, which were 68.96% and 68.64%, respectively, and chromosome 3 of Hwayeong and BLB, which were 61.2% and 71.2%, respectively (Figure 3).

### Line-specific SNP analysis

**Figure 4:**
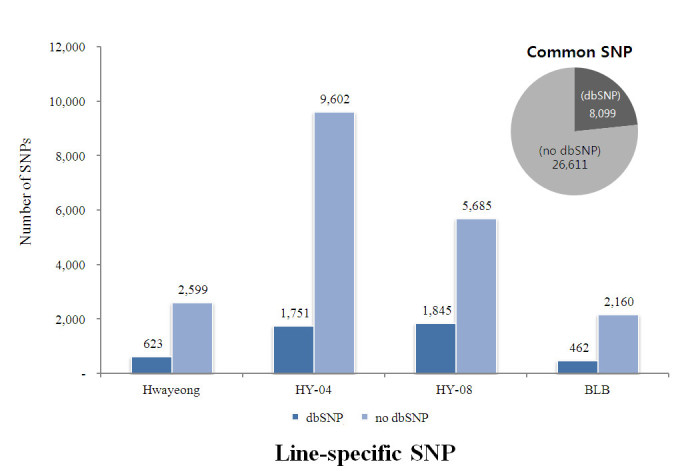
**Line-specific SNPs and common SNPs.** SNPs were classified as specific to Hwayeong and each of its progeny lines and SNPs common to them all. Line-specific SNPs and common SNPs were further classified into two groups. One group consisted of novel SNPs, which were not reported in the dbSNP (no dbSNP), and the others were listed in the dbSNP. In the graph, the x-coordinate and y-coordinate represent each chromosome and the number of SNPs, respectively.

lsSNPs unique to Hwayeong and each of its progeny lines (BLB, HY-04 and HY-08) were identified. These candidate SNPs have the possibility of being associated with a unique phenotype or agronomical trait in each cultivar or line. The lsSNPs were classified as those not previously reported in the dbSNP. Unique SNPs were detected in each of these lines. It was estimated that the portion of lsSNPs is 1 to 3% of the total SNPs (Table 4, Figure 4). The distribution of non-synonymous SNPs out of lsSNPs varied from each line. In Hwayeong, SNPs were distributed only on chromosomes 5, 7, 8, and 11 and similarly, in BLB they were detected on chromosomes 2, 5, 8, and 12. In both lines, the majority of non-synonymous SNPs were distributed on chromosome 8. In HY-04 and HY-08, however, there was a more even distribution among the chromosomes. They also had larger numbers of lsSNPs on chromosome 1 than Hwayeong and BLB. The HY-04 line had the highest number of the lsSNPs with 9,602 (3.4% of the total SNPs). The BLB, on the other hand, contained only 2,160 lsSNPs (1.0% of the total SNPs), which was the lowest among the four lines. Most SNPs were located in the intergenic regions, 2,300 SNPs (88.5% of lsSNPs) in Hwayeong to 7,972 SNPs (83.0% of lsSNPs) in HY-04 (Table 4). The number of lsSNPs detected in the coding region varied from 48 SNPs (1.9%) in Hwayeong to 346 SNPs (3.6%) in HY-04. The frequency of non-synonymous SNPs in the coding regions also was different among the accessions. Hwayeong contained 26 SNPs (1.0% of lsSNPs) while HY-04 had 214 SNPs (2.2% of lsSNPs) (Table 4). Also SNPs common to all four accessions were identified. A total of 34,710 SNPs were common to all four accessions. Of those, 8,099 SNPs were unique to only these four lines and were classified as not reported in the dbSNP (Figure 4).

**Table 4:** **Line-specific SNPs not reported in dbSNP**

	Region/type	Hwayeong		BLB		HY-04		HY-08	
		(#)	(%)	(#)	(%)	(#)	(%)	(#)	(%)
Location	Total lsSNP	2,599		2,160		9,602		5,685	
	Intergenic	2,300	(88.5)	1,821	(84.3)	7,972	(83.0)	4,990	(87.8)
	Intron	175	(6.7)	172	(8.0)	887	(9.2)	433	(7.6)
	5^′^ UTR	26	(1.0)	31	(1.4)	139	(1.4)	68	(1.2)
	CDS	48	(1.8)	73	(3.4)	346	(3.6)	96	(1.7)
	Non-synonymous	26	(1.0)	44	(2.0)	214	(2.2)	63	(1.1)
	Synonymous	22	(0.8)	29	(1.3)	132	(1.4)	33	(0.6)
	3^′^ UTR	43	(1.7)	49	(2.3)	229	(2.4)	85	(1.5)
	Exon	7	(0.3)	14	(0.6)	29	(0.3)	13	(0.2)
Type	Deletion	209	(8.0)	267	(12.4)	736	(7.7)	590	(10.4)
	Insertion	288	(11.1)	189	(8.8)	1,111	(11.5)	840	(14.8)
	Substitution	2,102	(80.9)	1,704	(78.8)	7,754	(80.8)	4,255	(74.8)

### Functional study

We analyzed the five genes that had the highest number of SNPs within a genic region in each of the five accessions. The genes *Os08g0205150* and *Os08g023640* 0 were included in the upper five SNP containing genes of Hwayeong and its progeny lines. Both genes have functions related to and including ATP binding, protein serine/threonine kinase activity, and protein amino acid phosphorylation. We also analyzed the SNP frequency in the top five genes in the three accessions developed from anther cultures. HY-04 harbored a total of 122 SNPs in one gene, *Os02g016400*, in which no SNP was found in Hwayeong. Only 50 coding SNPs (cSNPs) were located in the coding regions. Among these 50 cSNPs, six SNPs were detected as non-synonymous SNP (nsSNPs) and 44 SNPs were synonymous SNP. The lines BLB and HY-08 carried fewer SNPs, 13 and 14, respectively, in this gene. The difference in the number of SNPs between HY-04 and BLB or HY-08 is correlated with the difference in the number of SNPs in the coding region. Only four and five cSNPs are present in BLB and in HY-08, respectively. For the *Os07g0645700* gene, 54 cSNPs were detected on the CDS in HY-08 and there were 22 nsSNPs. BLB contained 40 cSNPs in the coding region but the number of nsSNPs was only three. Also HY-04 had five nsSNPs in this gene.

**Figure 5:**
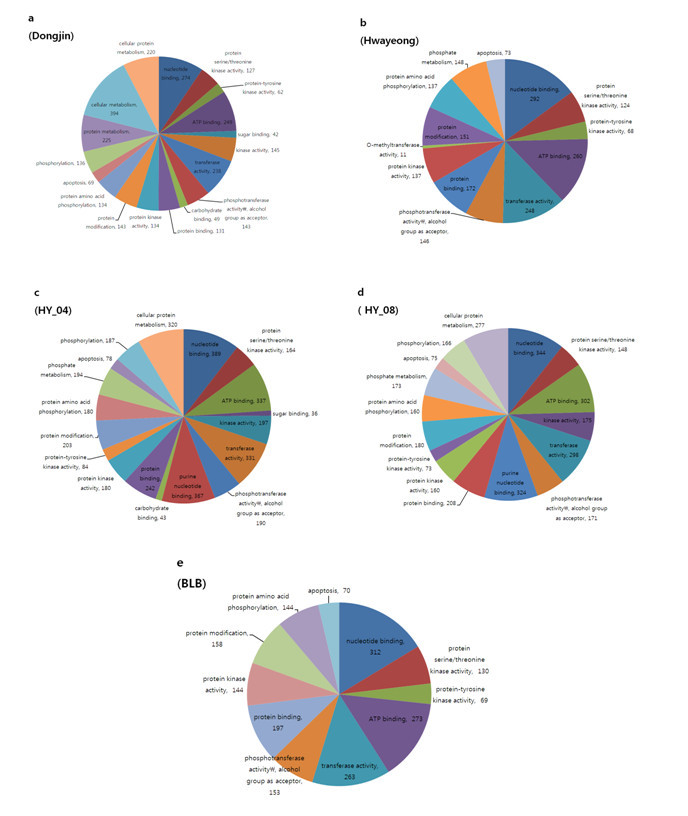
**Functional analysis of genes carrying non-synonymous SNPs.** Genes that contained one or more non-synonymous SNPs were separated into functional categories to obtain relationships between the gene’s function and potential SNPs by Gene Ontology.

To estimate the functional relationship of SNPs with genes in which SNPs reside, these genes were functionally classified based on GO. When we examined gene groups that carried one or more nsSNP, we discovered that all the accessions had plenty of SNPs in genes closely related to nucleotide binding (GO:0000166) and ATP binding (GO:0005524) (Figure 5). HY-04 and HY-08 especially showed that genes associated with purine nucleotide binding (GO:0017076) harbored many SNPs but this was not seen in the other accessions (Figure 5). In Hwayeong, 11 genes associated with the function of O-methyltransferase activity (GO: 0008171) had one or more SNP in the coding region, but the other four accessions did not have a SNP. HY-04 and HY-08 especially showed that genes associated with purine nucleotide binding (GO:0017076) and cellular protein metabolism (GO:0044267) possessed many SNPs in the coding region but Hwayeong and BLB did not appear to have these SNPs (Figure 5).

## Discussion

Anther culture systems have made a significant impact on plant breeding and genetics (Evans 1989; Sugimoto et al. 2000). Anther culture-derived plants are believed to undergo a spontaneous doubling of the haploid chromosomes of microsporocytes or callus cells. Therefore, anther culturing has been utilized to achieve rapid homozygosity and to enhance selection efficiency for important agronomical traits in plants. Also, like other tissue culture systems, it has been reported that a number of variants were generated among anther culture-derived plants, including rice (Bairu et al. 2011; Evans 1989; Roy and Mandal 2005; Yan et al. 1996). The progenies that were developed from anther culturing showed different types of variations from their mother plant, such as culm length, panicle length, and grain weight (Sohn et al. 1995; Yi et al. 1999). Therefore, genetic and breeding research with anther culture derived lines has been performed to obtain variation in important agronomic traits, and these lines are valuable genetic resources (Evans 1989; Schaeffer and Sharpe 1981). Even though the significance of anther culturing has been emphasized in terms of genetic variation, there is little information on the origin and extent of mutations derived during anther culturing. Most information has been obtained from the study of epigenetic and genetic activities of endogenous transposable elements (Barret et al. 2006; Kikuchi et al. 2003). In vitro, Kikuchi et al. (2003) showed that *miniature Ping* (*mPing*) elements, which is a new class of miniature inverted-repeat transposable elements, are activated in cells derived from anther cultures where mPing elements are deleted from original sites and reinserted into new loci. Barret et al. (2006) demonstrated that ZmTPA*Pong*-like in maize displayed homology with the transposase of *Pong*, and it could form part of a *Zea mays* element related to the rice *Pong* element. They also revealed somaclonal variations among plants regenerated from a doubled haploid line. Recently, it has been demonstrated that somaclonal variations result from newly induced mutations during the tissue culture process and not pre-exist in the plants before being cultured (Sato et al. 2011).

To estimate DNA polymorphisms between a mother plant and its descendants developed from anther culturing, we selected Hwayeong and three lines (BLB, HY-04, and HY-08) derived from Hwayeong via anther culturing. HY-08 and HY-04 have a high yielding ability and BLB has resistance to bacterial blight. These lines were subjected to whole-genome sequencing using a high-throughput sequencer. We also performed sequencing on Dongjin, which is an elite cultivar in Korea. All of the lines are in a japonica genetic background.

In the present study, the whole genome of five accessions was mapped to *Nipponbare* as a reference genome to discover genome-wide DNA polymorphisms. The uniquely mapped reads from these accessions covered > 95% of the reference genome, providing an average coverage of 54.6× across the genome (Table 1). Among the chromosomes, chromosome 5 had a high mapping ratio, > 99%, while chromosome 11 had the lowest ratio. A notable enrichment of significant structural variation which includes copy number variation (CNV) caused by large insertion, deletion or duplication have been identified within known R gene clusters in several crop species, such as soybean and rice (McHale et al. 2012; Yu et al. 2011). Therefore, it may be inferred that diverse structural variation was occurred on chromosome 11 of which dense genes or gene family were associated with disease resistance and immunity (The Rice Chromosomes 11 and 12 Sequencing Consortia 2005). The relationship between sequencing depth and efficacy in the comprehensive detection of SNPs is a key concern from the perspective of cost-effectiveness. Smith et al. (2008) reported that redundancy resulting from increasing the sequencing depth from 10× to 15× permits accurate and cost-effective detection of DNA polymorphisms using a Solexa analyzer. As mentioned above, we achieved the final effective mapping depth of > 54.6× coverage. Based on result of mapping reads, we detected the total of 1,154,063 DNA polymorphisms including 1,024,202 SNPs, 53,180 insertions and 76,681 deletions between the five accessions and the reference genome, with an average density of a SNP per 1.6 kb on *Nipponbare*. Dongjin bred by a conventional breeding method had a lower number of SNPs (0.64 SNP/kb) than Hwayeong and its progenies obtained from an anther cultivar (average density of 0.45 SNP/kb). SNPs were concentrated (> 50%) on chromosome 11 and 12 of Dongjin and on chromosome 8 and 11 of the anther culture lines and Hwayeong. Among Hwayeong and its progenies, HY-04 and HY-08 had more detected SNPs than Hwayeong and BLB. Particularly the ratio of SNPs on chromosome 1 was 5 times higher in HY-04 and HY-08 than Hwayeong. HY-04 and HY-08 exhibit high yield among them, which is a distinguishable agricultural trait from Hwayeong. Based on the study of Miura et al. (2011) and Vikram et al. (2011), QTLs for grain yield was identified on rice chromosome 1. We believe that this information is useful to find genes associated with important trait of both of them in further study. We classified the detected SNPs into two types, homozygous SNPs and heterozygous SNPs. Since Hwayeong and its progenies were lines driven by anther culturing and Dongjin was bred by selfing over a several generations, the detected SNPs were expected to be predominantly homozygous SNPs; however, 13% of the SNPs were heterozygous (Figure 1a). Accoding to the study of Pinson and Rutge (1993), they stated, it could be found in some mechanisms such as somatic tissues, mutation occurring during or after a spontaneous doubling event, fusion of genotypically different cells in chimeric callus, and abnormal meioses resulting in heterozygous diploid microspores. Although heterozygosity of SNPs in these accessions is difficult to explain, further studies will solve the cause of the heterozygosity in the near future.

If SNPs exert functional effects on phenotypic traits, they are most likely located in intra-genic regions. We therefore classified SNPs based on their genomic locations. Of the potential SNPs, 80% were located in intergenic regions and approximately 5% in coding regions. Hwayeong carried 10,244 SNPs (4.59% of the total) in coding regions. Of these, the number of nsSNP was 5,222 (2.34%). HY-04 contained 14,829 cSNPs (5.23%) of which 7,638 were nsSNPs (2.69%) (Figure 2).

HY-04 and HY-08 carried similar numbers of whole genome but the smallest number of cSNPs among the accessions. Among the 42,088 genes annotated with RAP-DB, 5,471 genes (13%) in Hwayeong contained one or more SNPs and the total number of SNPs was 39,029, which corresponds to 7.13 SNPs per gene (Table 3). Anther culture progenies of Hwayeong revealed slightly higher frequencies of SNPs than Hwayeong in genic regions.

The five genes that had the highest number of cSNPs in a genic region were investigated. As a result, variations were detected in genes related to immunity, such as apoptosis and signal transduction. All accessions included the genes *Os08g0205150* and *Os08g0236400*, which perform the functions of ATP binding, protein serine/threonine kinase activity and protein amino acid phosphorylation. However, the SNP frequency in each of the top five genes varied among accessions. HY-04 revealed 122 cSNPs in the *Os02g0164000* gene, while HY-08 and BLB had four cSNPs and three cSNPs, respectively. However, Hwayeong carried no SNPs in the same gene. The detection of DNA polymorphisms in this gene not only verified that HY-04 and HY-08 are anther culture-derivatives of Hwayeong but also revealed genetic differences between the progenies. The frequencies of cSNPs and nsSNPs, 54 and 21 on average, respectively, in the *Os07g06457001* gene were similar in HY-08 and Hwayeong. BLB, however, contained 40 cSNPs in the coding region of the *Os07g0645700* gene but the number of nsSNP in BLB was seven times lower than the other two accessions (3 nsSNPs).

Yamamoto et al. (2010) clarified the definition of the pedigree haplotypes of closely related rice cultivars to analyze conserved SNP regions between cultivars by means of genome-wide SNPs. In contrast to Yamamoto et al. (2010), we used lsSNPs to select candidate SNPs that could be associated with the phenotype of each cultivar. Based on the distribution of lsSNPs, we found certain regions and genes that were different between the mother line and its descendants and subsequently may influence the phenotype. HY-04 carried 9,602 lsSNPs, which was 3.4% of the total SNPs (Table 4). The distribution pattern of lsSNPs in the genome also was similar to that of SNPs in the whole genome. Greater than 83% of the lsSNPs were located in intergenic regions. HY-04 had the largest number of nsSNPs, 214, and Hwayeong had the smallest number of nsSNPs (Table 4, Figure 4). The lsSNPs identified in this study will provide valuable information used to isolate genes responsible for unique agronomical traits, which arise from almost identical lines generated by anther cultures. These lsSNPs will serve as molecular markers to map and clone genes that will distinguish its progenitor (mother line) and its anther culture siblings.

## Conclusions

The genetic diversity between the mother cultivar and its descendants obtained from anther cultivars was analyzed by revealing DNA polymorphisms, including single nucleotide polymorphisms, insertions and deletions among the five Korean rice accessions. The analysis estimated differences in genomic sequences between accessions using the frequency and distribution of SNPs in the genome, the five genes that had the largest number of SNPs in the coding regions and lsSNPs. The lsSNPs will be useful to select candidate SNPs that could have been associated with unique phenotypes or agronomical traits in each accessions. Furthermore, DNA polymorphisms will provide an invaluable resource to identify molecular markers and genes associated with diverse traits of agronomical importance.

## Methods

### Sample preparation and sequencing

Genomic DNA was extracted from five Korean rice accessions, including three anther culture lines (BLB, HY-04, and HY-08), their progenitor cultivar (Hwayeong), and a Korean elite japonica cultivar (Dongjin), and prepared following the manufacturer’s protocols (Illumina). Fragments of the library were paired-end sequenced using Illumina’s Hiseq 2000. The length of all sequences generated was 101 nucleotides. In Dongjin, we performed whole-genome resequencing by two massive parallel sequencing including Illumina Hiseq 2000 and 454 GS FLX. The raw reads that were high quality with Phred Quality Values > Q20 (ASCII Character Code +33) on basis of Sanger Quality were used to analyze genetic variations in five accessions. The “Q20” value indicates an accuracy of 99% for the base called.

### Reference database

#### Genomic data

The five Korean rice accessions belong to the japonica rice variety. Therefore, *Oryza sativa* L. cv. *Nipponbare* was used as the reference sequence (Pseudomolecules Build 5.0, http://rgp.dna.affrc.go.jp/E/IRGSP/Build5/build5.html, International Rice Genome Sequencing Project 2005). Information from RAP-DB (http://rapdb.dna.affrc.go.jp/) was constructed and annotated to analyze structure and gene function.

***dbSNP*** The NCBI’s SNP database (dbSNP) provides valuable information from whole-genome sequencing and Next Generation Sequencing (http://www.ncbi.nlm.nih.gov/projects/SNP/).

### Mapping of reads and SNP detection

A large number of paired-end reads were assembled on to genomic sequences of the japonica rice cultivar *Nipponbare* using CLC Assembly Cell (ver. 3.2.2, http://www.clcbio.com) with the following parameters: alignment mode, local; similarity, 95%; HSP coverage 100%; gap cost, 3; deletion cost, 3; and mismatch cost, 2. SNPs were detected by comparison alignment with the *Nipponbare* sequence as a reference. To classify whether mismatches were sequencing errors or genomic variations, parameters were set as follows: minimum depth, 30; minimum variant frequency, 35%; least mismatch count, 20; and homo/heterozygote fold change, 2. RAP-DB was utilized to locate the discovered SNPs. SNPs were annotated as genic and intergenic based on positional information from the genome. DNA polymorphisms in genic regions were classified as coding sequence (CDS), untranslated regions (UTRs), and introns. DNA polymorphisms in the coding region were separated into synonymous SNPs and non-synonymous SNPs by amino acid substitutions. Also, SNPs were classified into two types, homozygous and heterozygous SNPs, based on the mismatch frequency if more than two bases shared the identity position.

### Comparison between SNPs and dbSNP

To get the specific variation information, we compared the potential SNPs in four accessions with the dbSNP. As the reference SNP (refSNP) position information of *O. sativa* provided on dbSNP is based on genome build 3. We redefined the SNP position information based on build 5. To update the refSNP to genome build 5, we reconstructed the refSNP position information based on 4,521,605 refSNPs reported in dbSNP (Table 5). Our results show that 3,985,423 refSNPs (88%) were updated with unique positions in the genome sequence, while about 12% of the refSNPs positions could not be confirmed because they mapped to multiple locations or were not mappable (Table 6). We were able to successfully update to genome build 5 when considering approximately 12% of undefined rsSNPs had no information of unique genome positions in genome build 3. Using the redefined dbSNP, we analyzed whether the detected SNPs were novel SNPs or common SNPs already reported in the dbSNP.

**Table 5:** **Statistics of dbSNP**

chr	Number of rsfSNP	Build4.0 genome sequence		Build5.0 genome sequence	
		Number of mapping rsfSNP	Rate of mapping rsfSNP	Number of mapping rsfSNP	Rate of mapping rsfSNP
Total	4,521,605	3,985,629	0.881463	3,985,423	0.881418
	(5,418,373)	(4,481,743)	(0.827138)	(4,481,537)	(0.827100)
1	547,062 (12%)	481,321 (12%)	0.879829	481,344 (12%)	0.879871
2	463,395 (10%)	408,263 (10%)	0.881026	408,250 (10%)	0.880998
3	449,034 (10%)	402,287 (10%)	0.895894	402,289 (10%)	0.895899
4	367,590 (8%)	323,843 (8%)	0.880990	323,783 (8%)	0.880826
5	354,080 (8%)	314,864 (8%)	0.889245	314,857 (8%)	0.889226
6	379,978 (8%)	336,607 (8%)	0.885859	336,617 (8%)	0.885885
7	361,174 (8%)	315,166 (8%)	0.872615	315,185 (8%)	0.872668
8	355,927 (8%)	314,842 (8%)	0.884569	314,850 (8%)	0.884592
9	302,749 (7%)	268,656 (7%)	0.887389	268,632 (7%)	0.887309
10	286,270 (6%)	250,494 (6%)	0.875027	250,453 (6%)	0.874884
11	332,510 (7%)	288,612 (7%)	0.867980	288,529 (7%)	0.867730
12	321,836 (7%)	280,674 (7%)	0.872103	280,634 (7%)	0.871978
Multi^a^	697,672	472,089	0.676663	472,089	0.676663
NotOn^b^	199,096	24,025	0.12067	24,025	0.12067

a: reads which mapped in multiple regions.

b: reads which not mapped.

**Table 6:** **The number of rsSNP according to genome version**

Genome version	Number of mapping rsfSNP	Rate of mapping rsfSNP	Number of rsfSNP
Build 4.0	3,985,629	88.14%	4,521,605
Build 5.0	3,985,423	88.14%	

### Functional study

To estimate the functional relationship of the SNPs with genes, we performed the three analyses. First, the five genes that had the highest number of SNPs within a genic region were selected and the functions of genes were compared between each accession. Second, genes were functionally classified based on Gene Ontology (GO; http://www.geneontology.org/). Finally, the lsSNPs were classified as those not previously reported in the dbSNP. Unique SNPs were detected in each accession.

## Accession codes

The resequencing data from the five Korean rice accessions have been submitted to EMBL-EBI (http://www.ebi.ac.uk) under the accession numbers; Dongjin [ERP001605, ERP001678], Hwayeong [ERP001620], BLB [ERP001655], HY-04 [ERP001653], HY-08 [ERP001654].

## Authors’ information

IS, UH, GS, HS, HJ, JH, TH: Genomics Division, National Academy of Agricultural Science, Rural Development Administration, Suwon 441–707, Republic of Korea. CD: Department of Biochemistry, Gyeongsang National University, Jinju 660–701, Republic of Korea. GA: Department of plant molecular systems biotechnology and Crop biotech institute, Kyung Hee university, Yongin 446–701, Republic of Korea.

## Authors’ original submitted files for images

Below are the links to the authors’ original submitted files for images. Authors’ original file for figure 1 Authors’ original file for figure 2 Authors’ original file for figure 3 Authors’ original file for figure 4 Authors’ original file for figure 5 Authors’ original file for figure 6 Authors’ original file for figure 7 Authors’ original file for figure 8 Authors’ original file for figure 9 Authors’ original file for figure 10

## Abbreviations

refSNP: Reference SNP
lsSNP: Line-specific SNP
cSNP: SNP within coding region
nsSNP: Non-synonymous SNP
