# Supplementary figures and images for: SNP-based analysis of genetic diversity in anther-derived rice by whole genome sequencing
Source: Rice (N Y). 2013 Mar 14;6:6. doi: 10.1186/1939-8433-6-6 (PMC4883692; doi:10.1186/1939-8433-6-6)

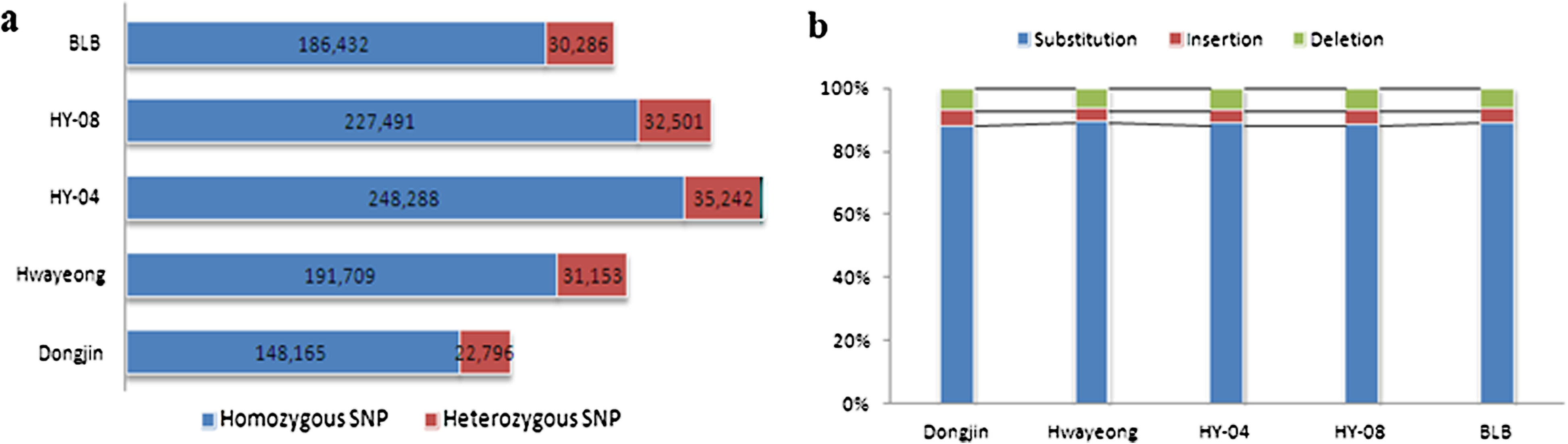

Supplement: Supplementary file 1 — Authors’ original file for figure 1 [file 12284_2012_44_MOESM1_ESM.tiff]

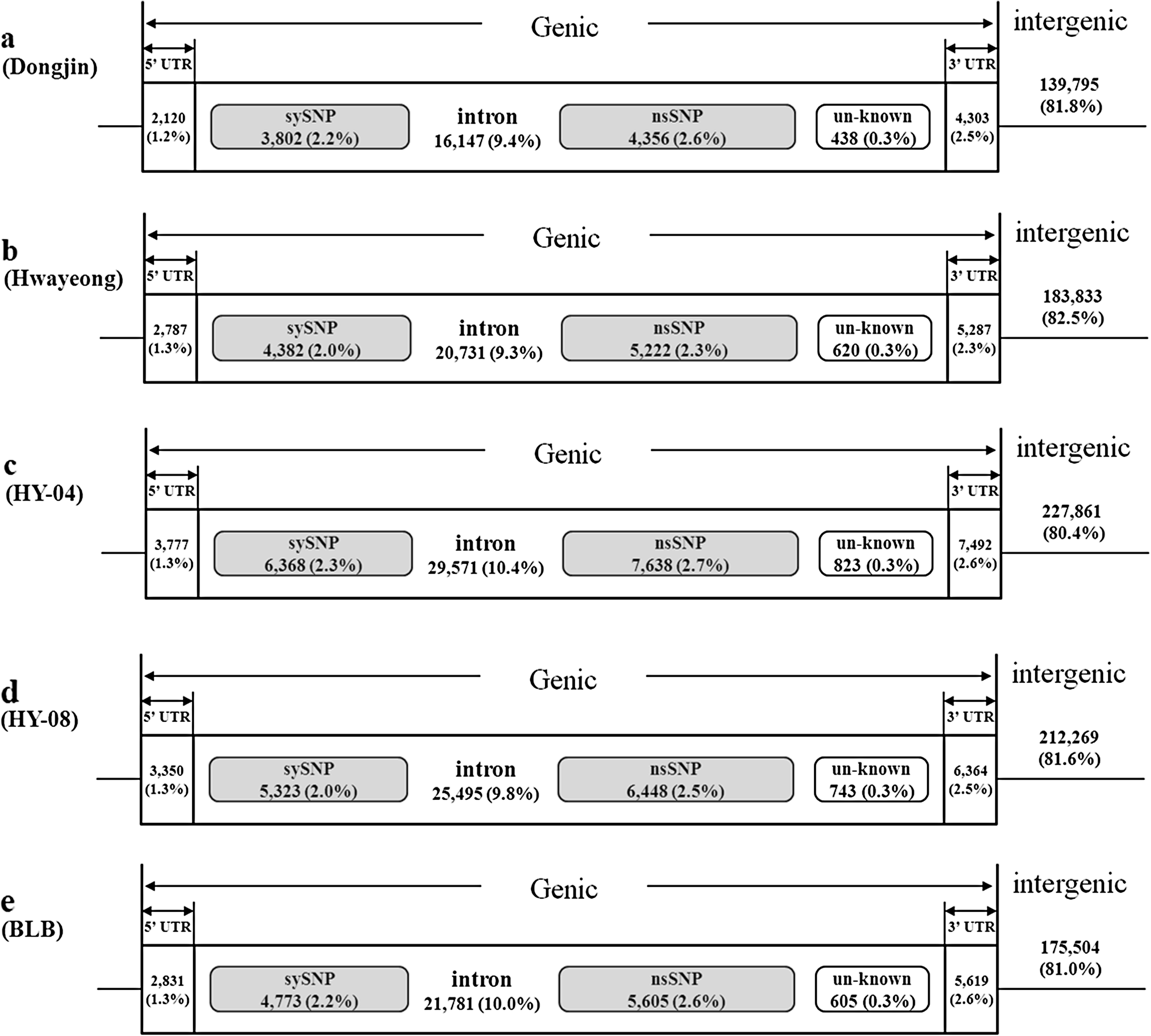

Supplement: Supplementary file 2 — Authors’ original file for figure 2 [file 12284_2012_44_MOESM2_ESM.tiff]

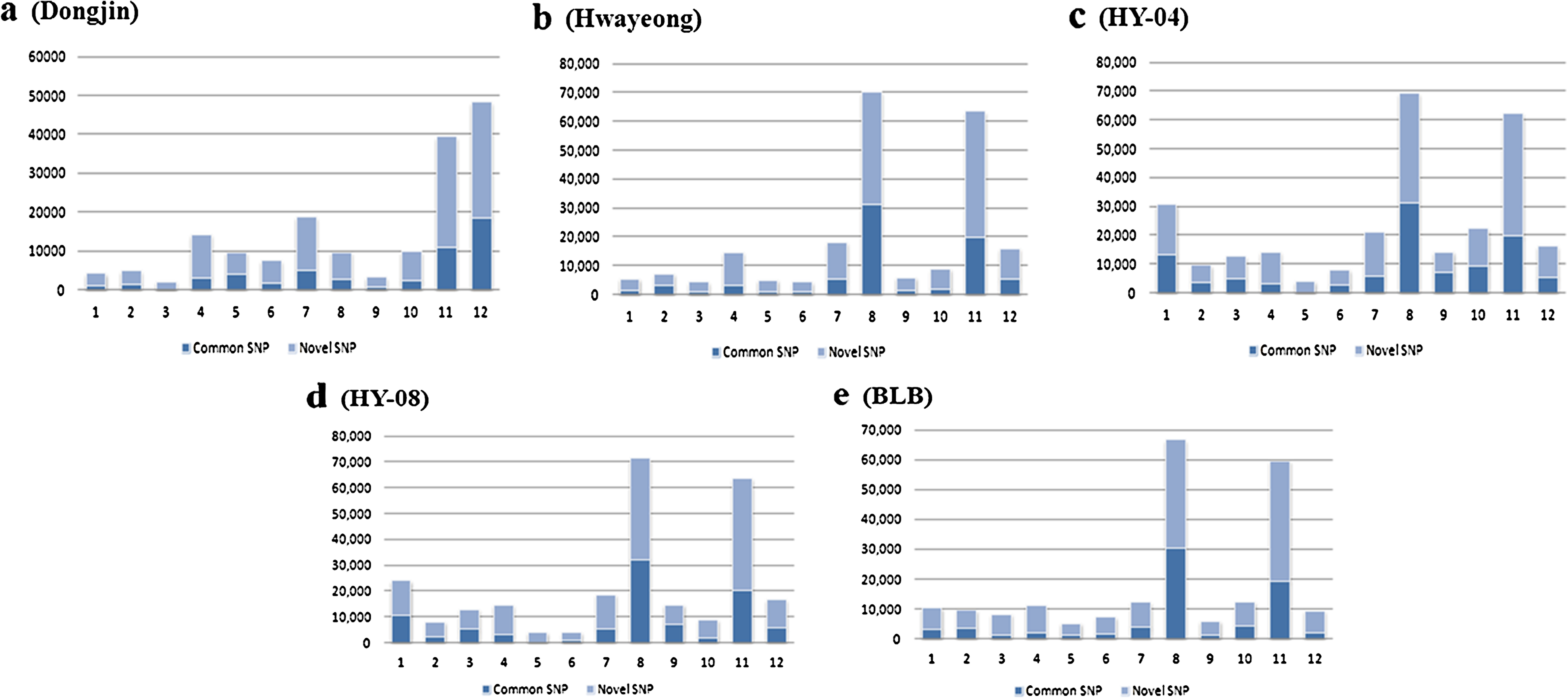

Supplement: Supplementary file 3 — Authors’ original file for figure 3 [file 12284_2012_44_MOESM3_ESM.tiff]

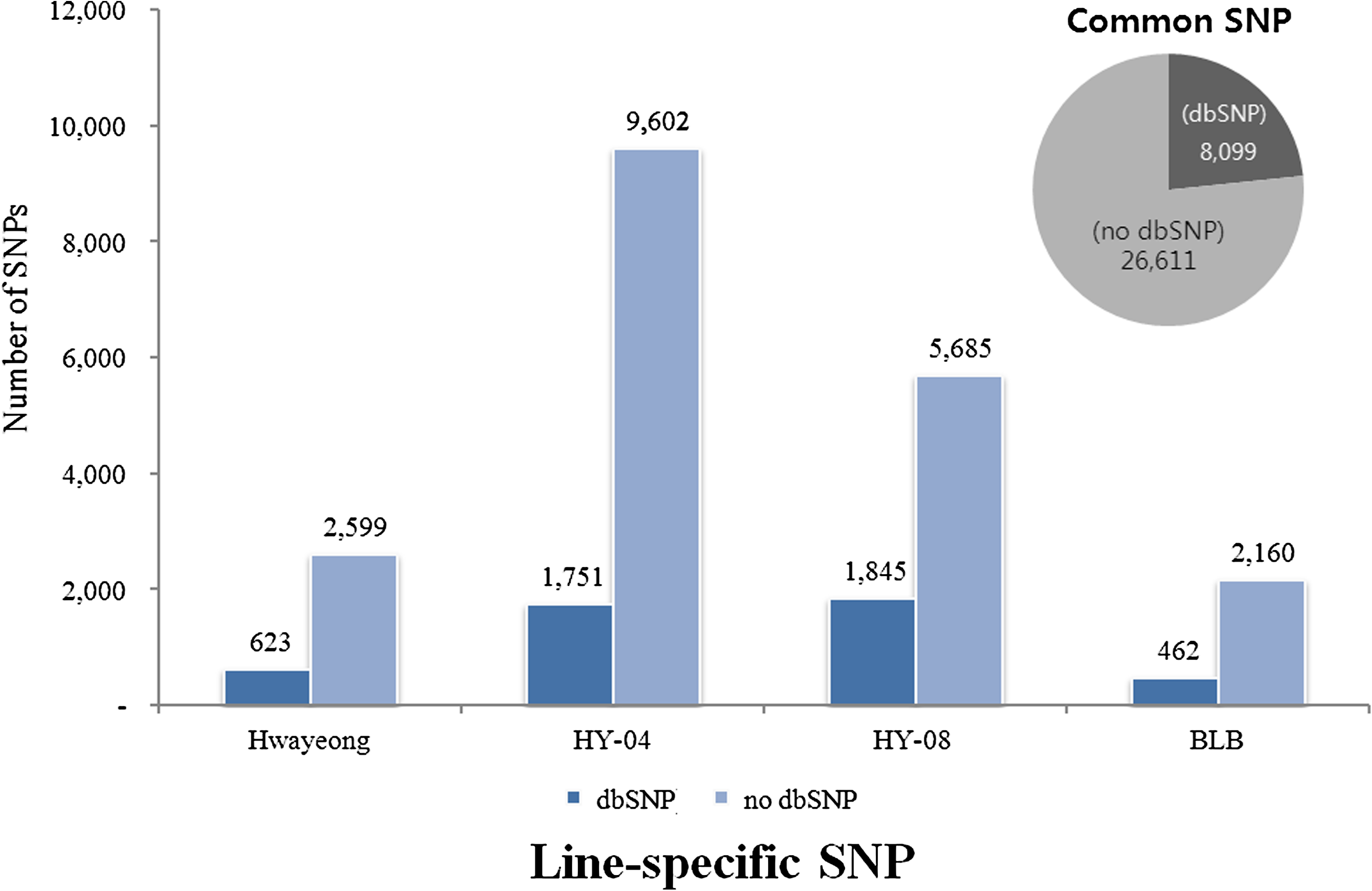

Supplement: Supplementary file 4 — Authors’ original file for figure 4 [file 12284_2012_44_MOESM4_ESM.tiff]

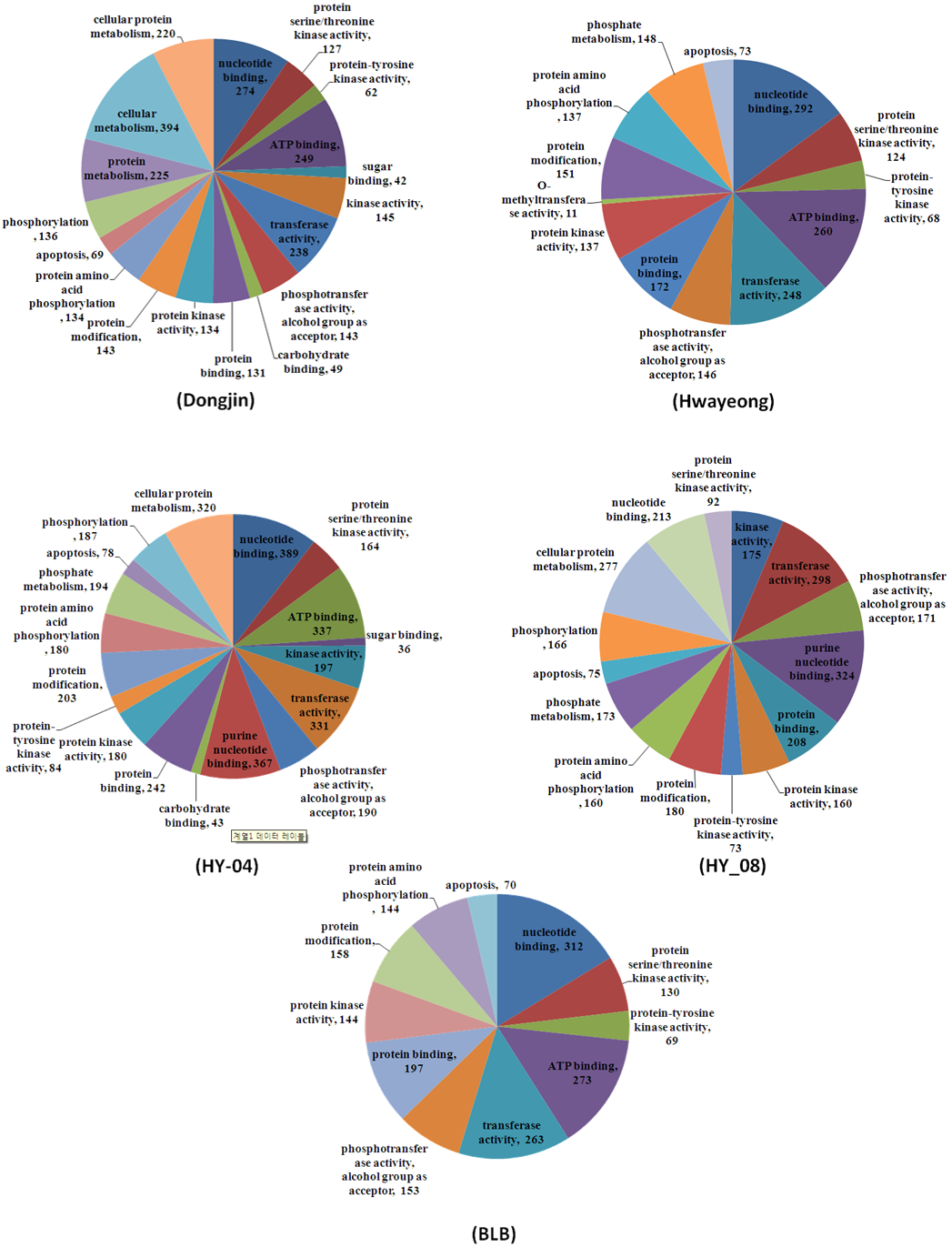

Supplement: Supplementary file 5 — Authors’ original file for figure 5 [file 12284_2012_44_MOESM5_ESM.tiff]

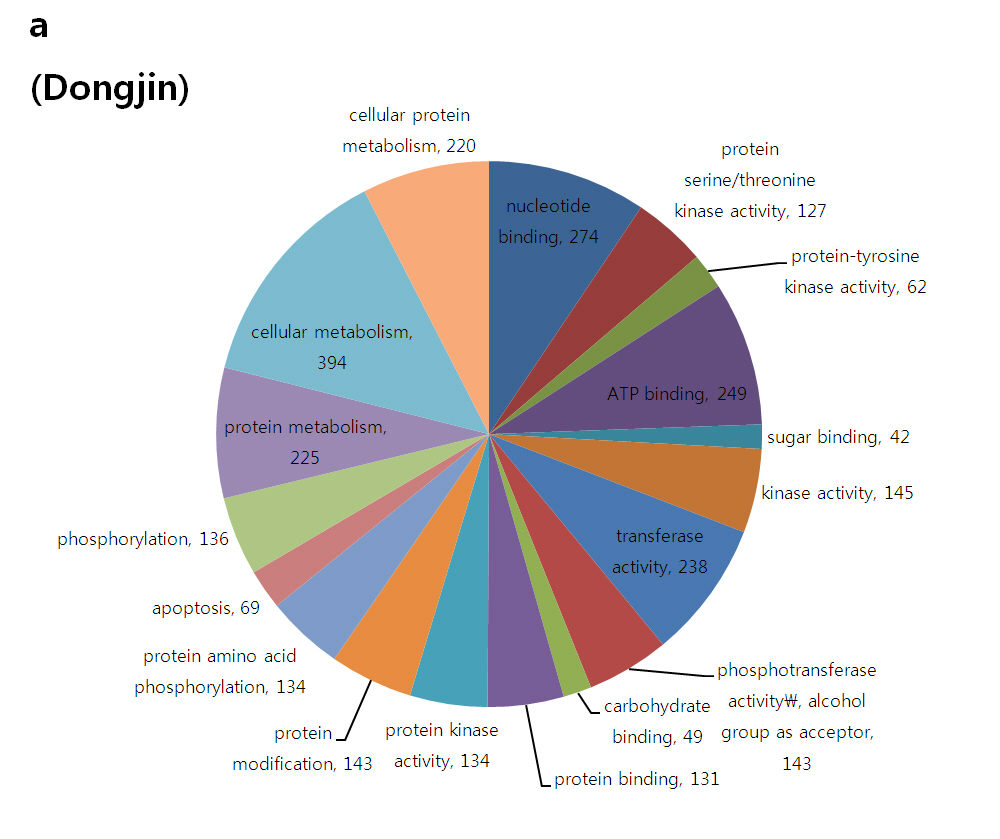

Supplement: Supplementary file 6 — Authors’ original file for figure 6 [file 12284_2012_44_MOESM6_ESM.png]

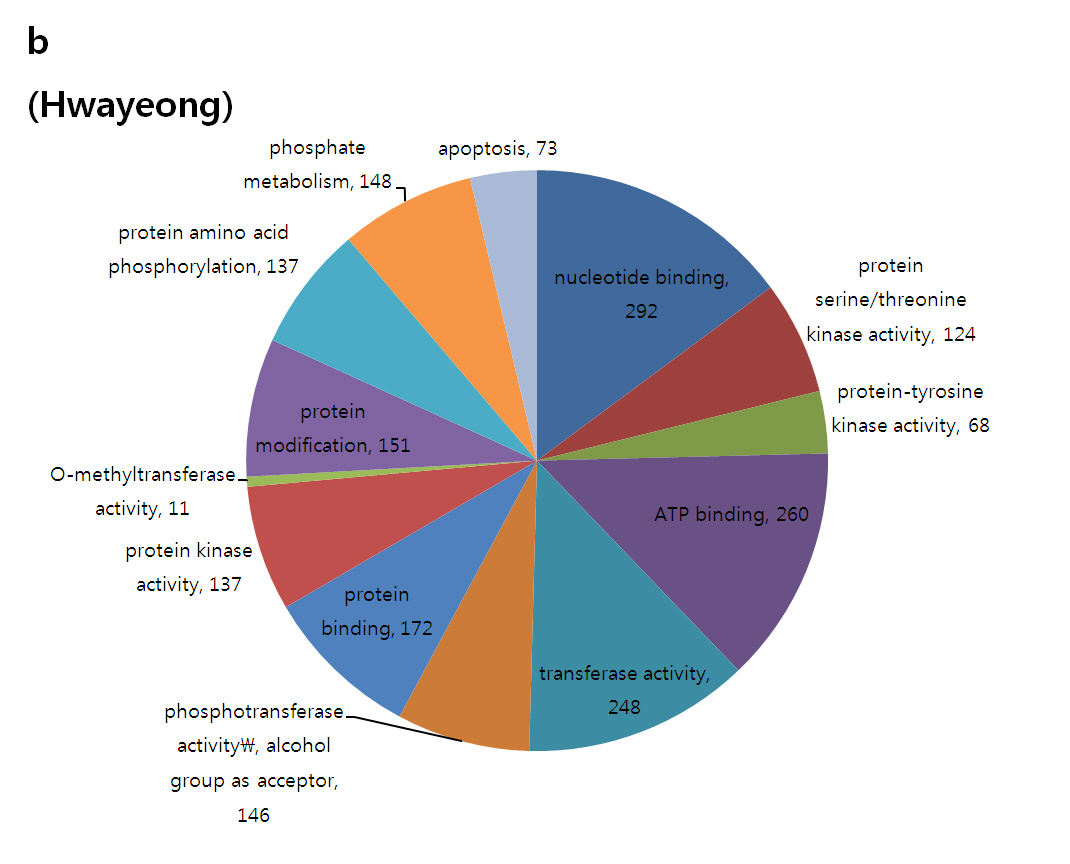

Supplement: Supplementary file 7 — Authors’ original file for figure 7 [file 12284_2012_44_MOESM7_ESM.png]

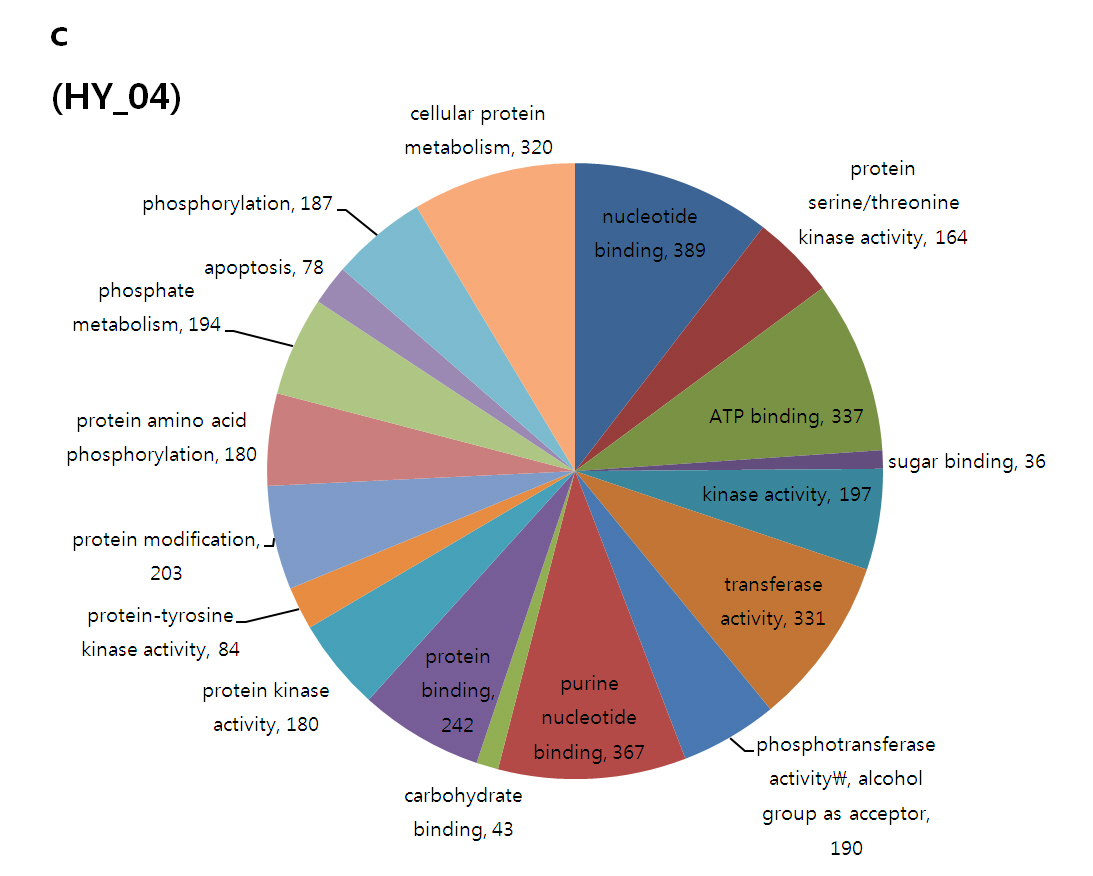

Supplement: Supplementary file 8 — Authors’ original file for figure 8 [file 12284_2012_44_MOESM8_ESM.png]

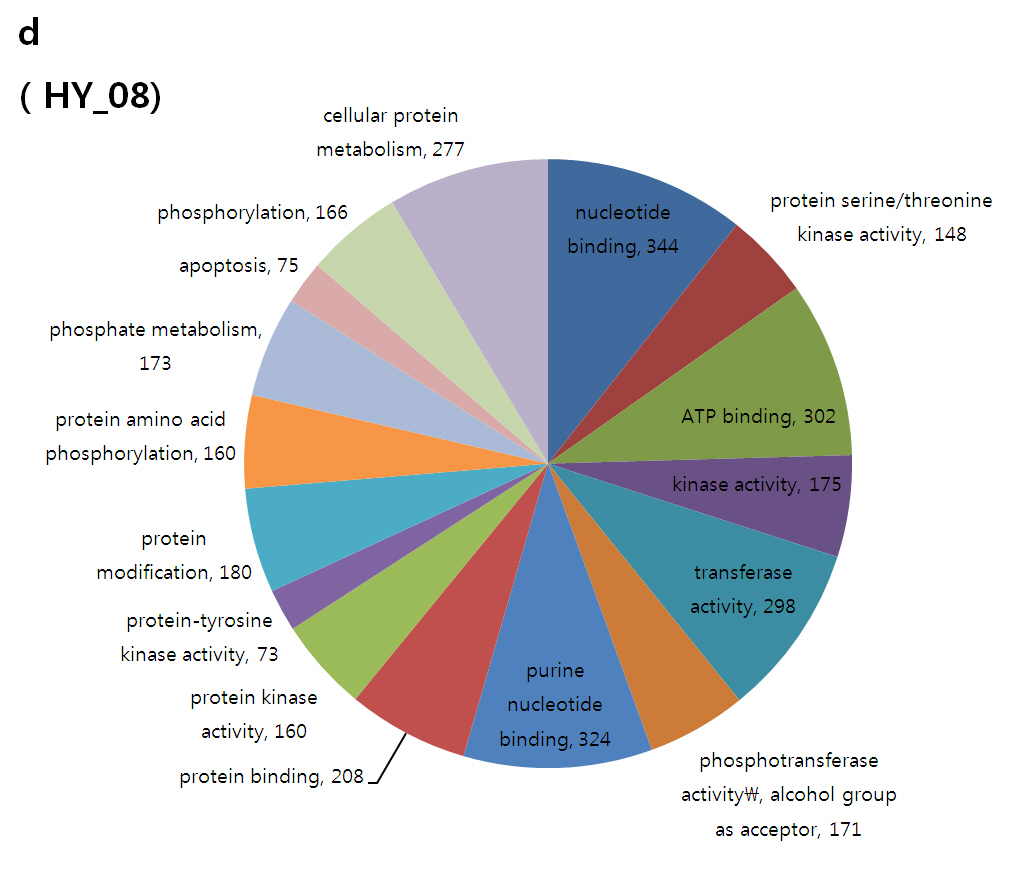

Supplement: Supplementary file 9 — Authors’ original file for figure 9 [file 12284_2012_44_MOESM9_ESM.png]

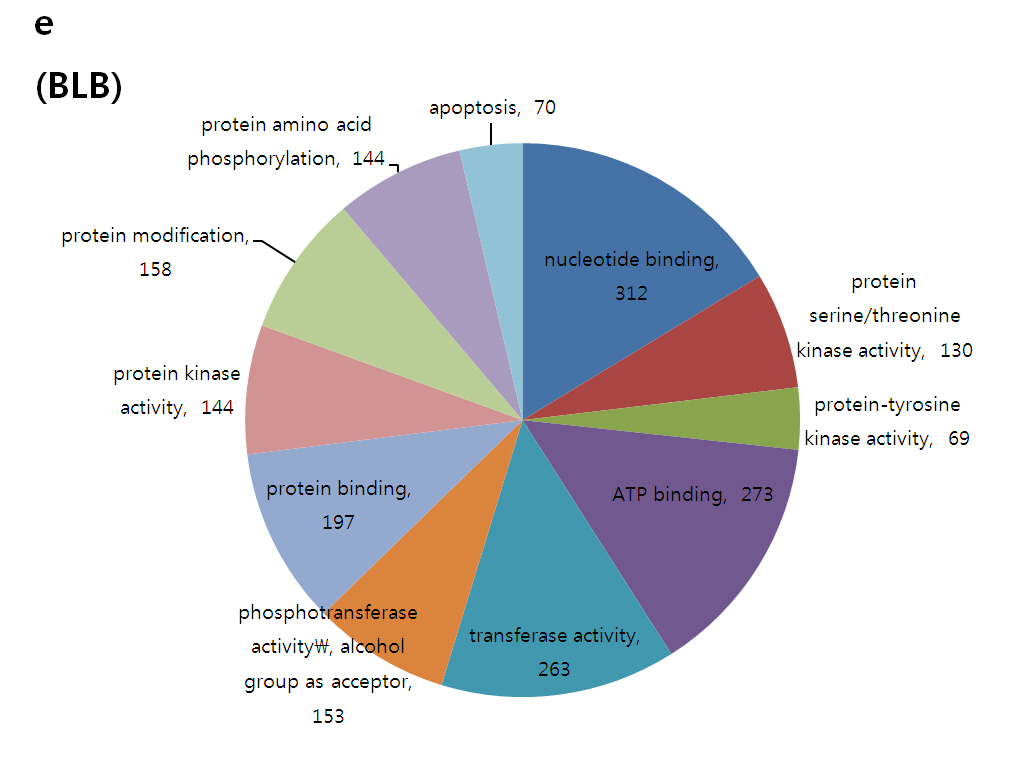

Supplement: Supplementary file 10 — Authors’ original file for figure 10 [file 12284_2012_44_MOESM10_ESM.png]
